# Neuroanatomical Signature of the Transition from Normal Cognition to MCI in Parkinson's Disease

**DOI:** 10.14336/AD.2024.0323

**Published:** 2024-03-23

**Authors:** Iman Beheshti, Jarrad Perron, Ji Hyun Ko

**Affiliations:** ^1^Department of Human Anatomy and Cell Science, Rady Faculty of Health Sciences, University of Manitoba, Winnipeg, MB, Canada.; ^2^PrairieNeuro Research Centre, Kleysen Institute for Advanced Medicine, Health Science Centre, Winnipeg, MB, Canada.; ^3^Graduate Program in Biomedical Engineering, Price Faculty of Engineering, University of Manitoba, Winnipeg, MB, Canada

**Keywords:** Anatomical MRI, accelerated brain-age, mild cognitive impairment, machine learning, Parkinson's disease

## Abstract

The progression of Parkinson’s disease (PD) is often accompanied by cognitive decline. We had previously developed a brain age estimation program utilizing structural MRI data of 949 healthy individuals from publicly available sources. Structural MRI data of 244 PD patients who were cognitively normal at baseline was acquired from the Parkinson Progression Markers Initiative (PPMI). 192 of these showed stable normal cognitive function from baseline out to 5 years (PD-SNC), and the remaining 52 had unstable normal cognition and developed mild cognitive impairment within 5 years (PD-UNC). 105 healthy controls were also included in the analysis as a reference. First, we examined if there were any baseline differences in regional brain structure between PD-UNC and PD-SNC cohorts utilizing the three most widely used atrophy estimation pipelines, i.e., voxel-based morphometry (VBM), deformation-based morphometry and cortical thickness analyses. We then investigated if accelerated brain age estimation with our multivariate regressive machine learning algorithm was different across these groups (HC, PD-SNC, and PD-UNC). As per the VBM analysis, PD-UNC patients demonstrated a noticeable increase in GM volume in the posterior and anterior lobes of the cerebellum, sub-lobar, extra-nuclear, thalamus, and pulvinar regions when compared to PD-SNC at baseline. PD-UNC patients were observed to have significantly older brain age compared to both PD-SNC patients (p=0.009) and healthy controls (p<0.009). The increase in GM volume in the PD-UNC group could potentially indicate an inflammatory or neuronal hypertrophy response, which could serve as a biomarker for future cognitive decline among this population.

## INTRODUCTION

Parkinson's disease (PD) is a complex neurodegenerative disorder that affects approximately 10 million people worldwide as of 2023 [1]. It is characterized by the progressive loss of dopaminergic neurons in the substantia nigra, which leads to the characteristic suite of motor symptoms like tremors, rigidity and bradykinesia [2]. PD is also associated with a range of non-motor symptoms, including cognitive impairment, which often impacts quality of life for both patients and caregivers [3, 4]. Approximately 50-80% of patients develop some kind of cognitive impairment after PD diagnosis [5]. Cognitive decline in people with PD is generally marked by a decrease in cognitive processing speed, challenges in executive function (such as planning, organizing, and prioritizing tasks), attention deficits impairment of working memory and compromised visuospatial abilities [6-8]. This may eventually progress to dementia, known as Parkinson’s disease dementia (PDD), which affects approximately 25% of people with PD [9].

Even though the cause of cognitive decline in PD is not rigorously understood, it is established that the degeneration of neurons responsible for dopamine production in the basal ganglia and the consequent disturbance of dopamine-related signaling pathways in the brain likely exert a significant impact [10, 11]. Additional factors that could potentially lead to a decrease in cognitive function in individuals with PD involve anomalies in diverse neurotransmitter systems, such as the acetylcholine and serotonin systems, as well as pathological protein deposits such as α-synuclein, and the effects of cerebrovascular ailments [8, 12]. Currently, Rivastigmine (a cholinesterase inhibitor; developed to treat Alzheimer’s disease symptoms) is the only FDA-approved (symptomatic) treatment for PDD. No treatment is available yet with the specific indication to treat PD with mild cognitive impairment (MCI).

Neuroimaging techniques have significantly improved our comprehension of the impacts of cognitive decline and its underlying mechanism in the brain of those with PD [13]. Several neuroimaging studies have reported that PD patients with cognitive decline have reduced gray matter volume in several major brain structures (e.g., left insular, left superior frontal and left middle temporal) compared to PD patients with intact cognition [14]. It is yet unknown whether these structural changes are specific to PD or associated with normal aging processes which is also characterized by atrophy in the similar brain regions. It is also unknown if these structural changes precede cognitive decline in PD, which may open a possibility of using magnetic resonance imaging (MRI) as a prognostic tool for cognitive decline in PD.

The amalgamation of neuroimaging methodologies with modern machine learning algorithms has proven to be an effective approach in brain studies. One particularly useful technique is brain age estimation. Brain age estimation allows one to estimate global brain health [15]. This method predicts an individual's “biological brain age” based on their brain’s anatomical or metabolism features and a supervised machine learning algorithm which may differ from the subject’s “chronological brain age” [16, 17]. This technique can help us to understand the effects of different factors (such as genetics, environmental factors, lifestyle, and neurological diseases) on relative rates of aging of the brain in a data-driven fashion [18, 19]. Furthermore, the application of brain age estimation can assist in identifying individuals who might be prone to age-related cognitive decline and/or neurodegenerative disorders prior to the onset of any clinical symptoms [18].

This technique has been widely applied throughout the literature in computational neuroimaging, and is most commonly applied studies utilizing MRI, however, fluorodeoxyglucose positron emission tomography (FDG-PET) is also used in the literature and has been shown to be a sensitive tool for detecting age-related changes in brain structure and function in a very wide variety of neurological disorders and neurodegenerative diseases [16]. Brain age estimation may be a useful tool for identifying PD patients at risk of cognitive decline and progression to dementia, which could allow personalized treatment plans[16]. To date, a limited number of studies have explored brain age in the context of PD [20, 21]. For example, the brain-predicted age difference (Brain-PAD) metric (difference between chronological and biological brain ages) has previously shown to be cross-sectionally correlated with both the Unified Parkinson’s Disease Rating Scale (UPDRS) III (motor symptom severity) and the Montreal Cognitive Assessment (MoCA; cognitive symptom severity) in PD patients [20, 21].

Brain age estimation remains a relatively new biomarker within the computational neuroimaging community, and so there still exist knowledge gaps in applying it across the field, such as in understanding the association between Brain-PAD in PD patients and their cognitive status. Our present work aims to address exactly this question. More specifically, we aim to uncover the neuroanatomical signature of incipient cognitive decline in PD patients who are cognitively healthy at baseline from the PPMI database. We used multiple biomarkers based upon MRI studies of the brain, specifically voxel-based morphometry (VBM), deformation-based morphometry (DBM) and cortical thickness measurements. We are specifically concerned with cognitively unstable PD patients who progressed from normal cognition at baseline to MCI within a five-year interval (PD-UNC). We also evaluated our brain age estimation hypothesis and measured Brain-PAD in our study cohorts. We hypothesize that PD-UNC will have greater Brain-PAD than PD-SNC, indicating that accelerated aging of potentially specific brain structures may precede cognitive decline in PD.

## MATERIAL AND METHODS

### Participants and MRI acquisition

Data for this study was provided by the Parkinson's Progression Markers Initiative (PPMI). 373 total subjects with PD were available who underwent T_1_-weighted MRI studies. Baseline MRI scans, demographic characteristics (i.e., age, education, onset age and disease duration) and clinical measurements were downloaded from the PPMI website in September 2022 (Table 1). The cognitive condition of the individuals with PD was assessed by analyzing the “*cogstate*” and “*MCI test score*” variables as documented in the PPMI dataset. In the PPMI study cognitive status is determined by evaluating different key domains: attention, memory, orientation, executive abilities and language. Dementia was identified by functional impairment in more than one cognitive domain, a decline from pre-morbid levels (i.e., pre-PD) and significant impact of impairment in daily life. MCI was similarly characterized by impairment in at least one cognitive domain, a decline from pre-morbid function, but crucially was distinguished by a lack of notable effect on activities of daily functioning or independence. Detailed information regarding cognitive status assessment in the PPMI can be found at https://www.ppmi-info.org. The "*cogstate*" variable for each subject was tracked over the course of the five-year follow-up and any missing time points were interpolated for each subject. We identified 244 patients with PD appropriate to our study which were divided into 2 groups based on their cognitive statues:

- PD-SNC subjects (N = 192): PD patients with stable normal cognition (SNC) at baseline who maintained this status during the five-year follow-up.
- PD-UNC subjects (N = 52): PD patients with unstable normal cognition (UNC) who had normal cognition at baseline but developed MCI within the five-year follow-up.

The accuracy of cogstate was verified by comparing the MCI test scores if available.

For both groups, annual cognitive assessments over a 5-year time span (after baseline) were used to define the cognitive status for our study. MRI scans were acquired at baseline. In the PD-UNC group, the conversion from normal cognition to MCI occurred from 1 to 5 years after baseline with a mean of 3.23 (±1.32 years). PD patients showing any fluctuations in their cognitive status (i.e., reverters) were not included in the study. All PD patients were unmedicated at baseline and free from any impairment in instrumental activities (e.g., shopping, using the telephone, preparing meals, using transportation, housekeeping, managing medication(s), managing finances, and participating in hobbies) of daily living (IADLs) [22].

**Table 1:** details of key domains used for determining cognitive status in PPMI dataset [55].

Cognitive Function	Description	Neurological Test
**Attention and working memory**	Ability to sustain and direct attention; lapses	Letter number sequencing (LNS)[56]
**verbal learning/memory**	Registration, recall of recent events or important dates; new learning ability; misplacement of items; forgetting items	Hopkins verbal learning test revised (HVLT); Hopkins Verbal Learning Test-Revised (HVLT-R)[57]
**Orientation/Visuospatial judgment**	Forgetting appointments; estimating time; spatial or geographical orientation	Benton judgment of line orientation (BJLO)[58]
**Executive Abilities/processing speed**	Reasoning ability; making decisions; following instructions; difficulty with calculations	Symbol digit modalities test (SDM) [59]
**Language/verbal ability**	Word-finding problems; problems with naming or comprehension	Semantic fluency (SF)[60]

### Standard protocol approvals, registrations, and patient consents

This study was approved by the Health Research Ethics Board of the University of Manitoba. All PPMI sites were approved by an ethical standards committee prior to the commencement of the study and all participants gave written consent before taking part. Further details regarding PPMI protocol approvals, registrations and patient consents can be accessed at: https://www.ppmi-info.org/.

### MRI preprocessing

The T1-weighted MRI scans were preprocessed using the CAT12 toolbox (http://www.neuro.uni-jena.de/cat/), which is an extension of the Statistical Parametric Mapping (SPM12, https://www.fil.ion.ucl.ac.uk/spm/software/spm12/) software package. The pre-processing parameters were considered with the default settings. The technical details of VBM-based preprocessing have been described in [23]. Briefly, the T1-weighted MRI scans were corrected for bias-field distortions, non-brain tissues were removed, and the images were segmented into gray matter (GM), white matter (WM), and cerebrospinal fluid (CSF) images. Jacobian determinant (JD) images were also generated. The images were then normalized to MNI-space using the diffeomorphic anatomical registration through exponentiated Lie algebra (DARTEL) algorithm. The normalized GM, WM, CSF and JD images were smoothed using an isotropic Gaussian smoothing kernel with a full-width at half maximum (FWHM) of 8x8x8 mm^2^. GM and WM density images were used in subsequent VBM analysis, and the JD images were used for DBM analysis. The quality of the images was evaluated using the "Check Homogeneity" feature within the CAT12 toolbox, in addition to visual evaluation. Total intracranial volume (TIV) and total GM, WM, CSF volumes per subject were also computed with the CAT12 toolbox. Cortical thickness (CT) measurements were obtained using the CAT12 toolbox by employing the Desikan-Killiany-Tourville (DKT) atlas. This atlas is composed of 34 pre-defined regions of interest (ROIs) in each cerebral hemisphere that were utilized to assess cortical thickness [24]. For brain age estimation, GM, WM and CSF images were smoothed using an isotropic Gaussian smoothing kernel with a FWHM of 4x4x4 mm^3^, as suggested in [25], and then resampled to an 8-mm isotropic spatial resolution, which resulted in 3,747 voxels per volume.

### Brain-age prediction model

The details of our brain age prediction model are described elsewhere [26]. As an overview, we train our brain age prediction model using data from three datasets of healthy individuals: Information eXtraction from Images (IXI) dataset (http://brain-development.org/ixi-dataset/), Open Access Series of Imaging Studies (OASIS; https://www.oasis-brains.org/) and PPMI (www.ppmi-info.org). IXI includes 563 healthy control (HC) participants, OASIS 1 includes 313 HC participants and 120 participants diagnosed with Alzheimer's disease, and PPMI is composed of a group of participants who were diagnosed with PD (N = 373, at baseline) and a control group of HC participants (N = 198, at baseline). In total we had access to 1,054 T1-weighted (T1w) MRI scans from HCs were included in this study and all other subjects were not included. These HC subjects were then randomly divided into two main cohorts: a training set (90% of HCs; *N_train_* = 949, mean age ± SD: 49.75 ± 18.96, age range: 18-94, 54% female) and a validation set (i.e., 10% of HCs; *N_test_* = 105, mean age ± SD: 48.62 ± 19.14, age range: 18-93, 53% female). All HCs were free from any signs of cognitive impairment or neurological disorder according to database criteria. The validation set of HC subjects (N = 105) was used as a reference for brain age and volumetric comparison analysis.

The brain age estimation model consists of a support vector regression (SVR) algorithm with a linear kernel in MATLAB r2020b (The Mathworks, Natick, MA, USA). Structural brain features (i.e., GM, WM and CSF voxel intensities, TIV and total GM WM and CSF brain volumes) were fed into the SVR along with scanner vendor, field strength and sex as independent variables. Chronological age was the dependent output variable of this model. Mean absolute error (MAE), root mean square error (RMSE) and coefficient of determination (R^2^) were used to assess model accuracy in training and validation. The prediction accuracy of the training set was computed using a 10-fold cross-validation strategy. Brain-PAD was computed as a mean with a standard deviation (SD) and bias adjustment was implemented as described in [27]. The entire training set (*N_train_* = 949) was used to build the final prediction model and was later evaluated on the independent test data.

### Statistical analysis

SPM12 was used to perform ANCOVA analysis on the processed GM, WM and JD images to identify any morphological differences among test groups. As our focus was on differences between the PD-SNC and PD-UNC groups, the HC group was not considered to be of interest at this stage. Sex, age and TIV of the subjects were considered as covariates in the VBM analysis, whereas only sex and age were considered for the DBM analysis. The analysis was repeated to ensure that the VBM/DBM results were not affected by baseline clinical symptom severity (anxiety, GDS, MoCA, and UPDRS-III), we conducted VBM/DBM analyses via t-test in SPM12, incorporating age, sex, and TIV as covariates, and subsequently repeated the analyses with these additional factors included as covariates alongside age, sex, and TIV.

The peak-level *p*-value threshold was adjusted to <0.001 (uncorrected) and clusters with *q*<0.05 (cluster-level correction for false discovery rate, FDR) were considered significant.

Difference in cortical thickness measurements between the PD-SNC and PD-UNC groups were compared for each brain region via two-tailed general linear models (GLMs) with age and sex as covariates. All statistical analyses were performed in MATLAB. FDR was applied to correct for multiple comparisons. The mean Brain-PAD and ROI-based brain volumes among the test groups (i.e., HC, PD-SNC and PD-UNC) were examined using analysis of covariance (ANCOVA) with sex and age as covariates, then followed by post-hoc Bonferroni tests if applicable to examine the direction of group differences. An independent *t*-test was used to investigate the mean Brain-PAD between PD-SNC vs. PD-UNC. A significance threshold of *q*<0.05 was considered significant for all statistical tests.

The correlation between continuous variables was assessed using the Pearson correlation test, while the Spearman rank correlation test was used for discrete variables. To ensure a fair and unbiased comparison among groups in volumetric analyses, we performed regression to remove the effects of age, sex and TIV by referencing our independent group of healthy controls (N=105). To account for variations in demographic and clinical variables between PD-SNC and PD-UNC, we used propensity score matching (available in Python: https://pypi.org/project/psmpy/ [36]) to identify a set of matched PD-SNC patients (PD-SNC*, N=52) in terms of all baseline demographic and clinical variables presented in Table 2.

**Table 2:** Clinical demographics and brain-PAD results of PD patients included in this study.

Characteristics	HC	PD-SNC	PD-UNC	PD-SNC	PD-UNC	PD-SNC	PD-UNC
		Baseline		At time of Conversion		Last record	
**N (male %)**	105 (74%)	192 (61.95%)	52 (71.15%)	192 (61.95%)	52 (71.15%)	192 (61.95%)	52 (71.15%)
**Demographics**	Mean (SD)	Mean (SD)	Mean (SD)	Mean (SD)	Mean (SD)	Mean (SD)	Mean (SD)
**Age, years**	48.62 (19.14)	58.41 (10.05)	64.33 (7.81)***	n.a	n.a	n.a	n.a
**Onset age, years**	n.a	56.34 (10.17)	62.4 (7.91)***	n.a	n.a	n.a	n.a
**Age at PD diagnosis, years**	n.a	57.85 (9.99)	63.81 (7.58)***	n.a	n.a	n.a	n.a
**Education, years**	n.a	15.80 (2.70)	15.06 (2.52)	n.a	n.a	n.a	n.a
**Disease duration, months**	n.a	6.67 (6.76)	6.18 (6.72)	n.a	n.a	n.a	n.a
**Motor symptoms**							
**UPDRS-III (total)**	n.a	28.59 (12.08)	33.18 (13.31)*	n.a	61.77 (18.62)	42.99 (16.39)	63.30 (21.95)***
**UPDRS-III (total rigidity)**	n.a	3.61 (2.46)	3.46 (2.60)	n.a	5662 (3.12)	4.45 (3.06)	5.94 (3.65)**
**UPDRS-III (total tremor)**	n.a	4.09 (2.77)	5.10 (3.84)	n.a	6.7 (5.3)	3.91(3.66)	4.80 (4.98)
**Cognitive symptoms**							
**MoCA**	n.a	27.27 (2.06)	26.56 (2.93)	n.a	24.51 (3.95)	27.69 (2.14)	24.15 (4.45)***
**LNS**	n.a	11.24 (2.57)	9.63 (2.72)**	n.a	8.98 (3.17)	10.88 (2.71)	8.67 (2.99)***
**BJLOT**	n.a	13.21 (1.88)	12.30 (2.47)**	n.a	11.32 (3.05)	12.79 (2.09)	11.09 (2.94)***
**SDM**	n.a	44.47 (9.03)	38.78 (8.86)**	n.a	34.19 (11.22)	44.23 (10.41)	32.84 (12.54)***
**SFT**	n.a	51.04 (10.95)	45.88 (9.69)**	n.a	44.38 (10.80)	52.30 (11.85)	43.26 (11.82)***
**Mood**							
**Anxiety**	n.a	62.99 (16.83)	68.25 (17.62)	n.a	70.0 (19.30)	60.13 (17.18)	71.88 (19.25)***
**GDS**	n.a	1.87 (2.06)	2.52 (2.40)	n.a	3.36 (2.72)	2.05 (2.26)	3.75 (2.74)***
**Medicine**							
**LEDD**	n.a	0	0	n.a	757 (577)	633 (427)	844 (591)

BJLOT: Benton judgment of line orientation score; GDS: Geriatric Depression Scale; HC: healthy control; LEDD: Total Levodopa Equivalent Daily Dose; LNS: Letter number sequencing; MoCA: Montreal Cognitive Assessment; PD: Parkinson diseases; SDM: Symbol digit modalities test; SFT: Semantic Fluency Total Score; UPDRS: Unified Parkinson Disease Rating Scale; N, number of subjects; n.a, not available; The HC group is the validation set that we used as an independent set for brain-age comparison (section 2.4). The significance values were reported as the results of a t-test for continuous variables and a Chi-2 test for categorical variables between the PD-UNC and PD-SNC groups after FDR correction for multiple comparisons, which was performed within each cluster (*P < 0.05, **P < 0.001, ***P < 0.0001). All HCs were free from any signs of cognitive impairment or neurological disorder according to database criteria.

## RESULTS

### Clinical and demographical characteristics

Both educational level and disease duration were similar between PD-UNC and PD-SNC (*q* > 0.10, FDR-corrected). The PD-UNC group had significantly greater age, age of onset and age at PD diagnosis than PD-SNC subjects (*q* < 0.001, FDR-corrected). This observation aligns with previous research that also found a statistically higher age and age at PD diagnosis in the PD-UNC group compared to the PD-SNC group [28].

There were no statistically significant differences in measures of disease severity (i.e., UPDRS-III totals, total rigidity and total tremor), cognitive symptoms (i.e. MoCA) or mood-related symptoms (anxiety, geriatric depression scale or GDS) between the two groups in our sample (*q* > 0.07, FDR-corrected; Table 2). Anxiety scores were calculated using the Parkinson Anxiety Scale (PAS), which is a 12-item scale rated on three subscales: persistent anxiety, episodic anxiety, and avoidance behavior. The PAS is rated by either observers or patients [29]. The demographic information of HC was described in Method section (2.4) and elsewhere [26].

To ensure comparability across different levels of clinical symptom severity and demographic characteristics in our analysis, we employed the propensity score matching technique to identify 52 PD-SNC patients (PD-SNC*). We verified that there were no significant differences between the PD-SNC* group and the PD-UNC group in terms of baseline age, age of onset, years since onset, age at PD diagnosis, level of education, disease duration, total UPDRS-III, UPDRS-III rigidity, UPDRS-III tremor, MoCA score, Letter Number Sequencing (LNS), Benton Judgment of Line Orientation (BJLOT) score, Symbol Digit Modalities Test (SDMT), Semantic Fluency Total Score (SFT), mood, and anxiety (*p* > 0.32).

**Figure 1.**
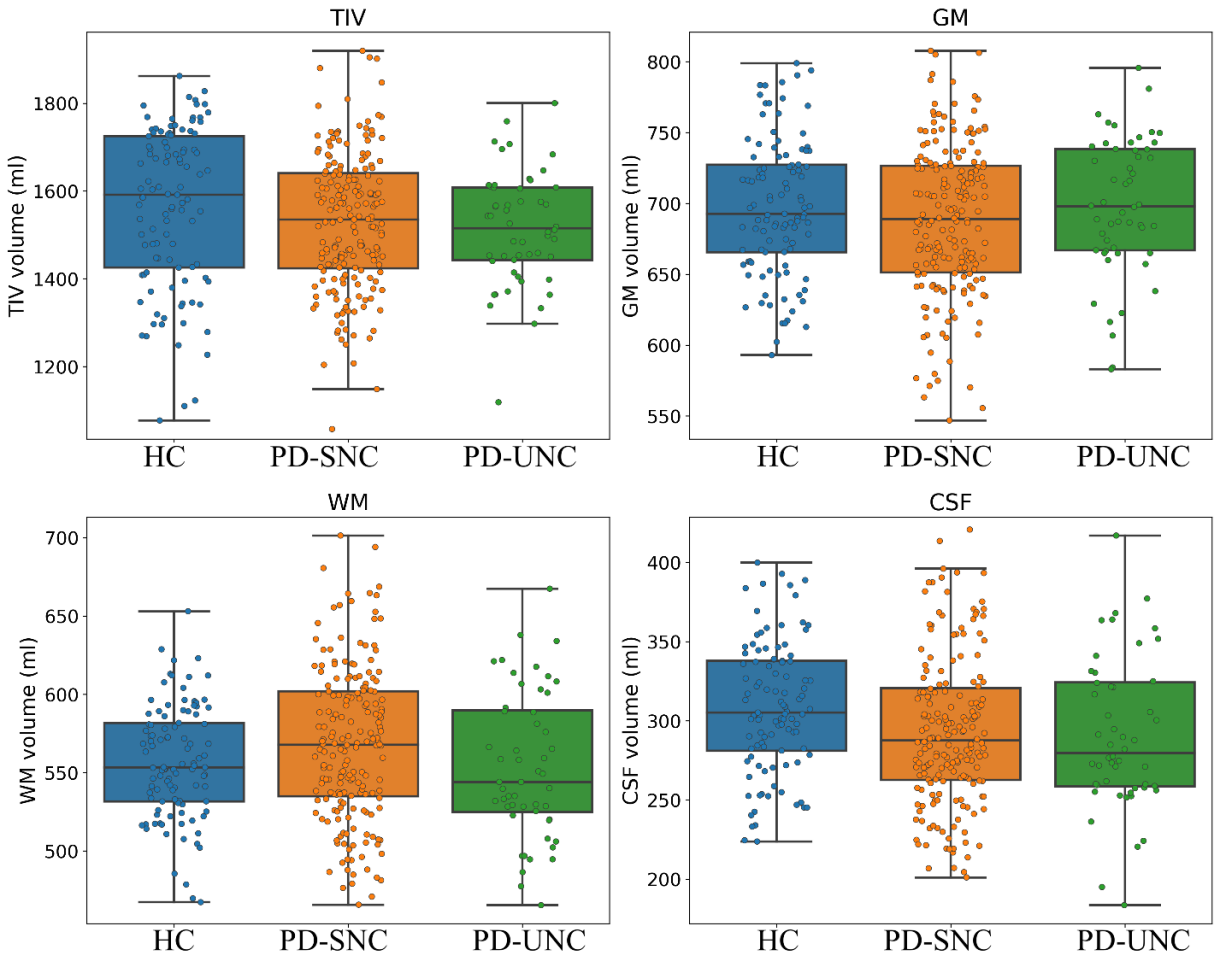
**The boxplots illustrating GM, WM, CSF, and TIV volumes among independent test groups (i.e., HC, PD-SNC, and PD-UNC)**. Age, sex, and TIV effects were removed from GM, WM, and CSF volumes through regression referencing the HC group.

### Brain structural analysis: Brain volumes, VBM and DBM

**Figure 2.**
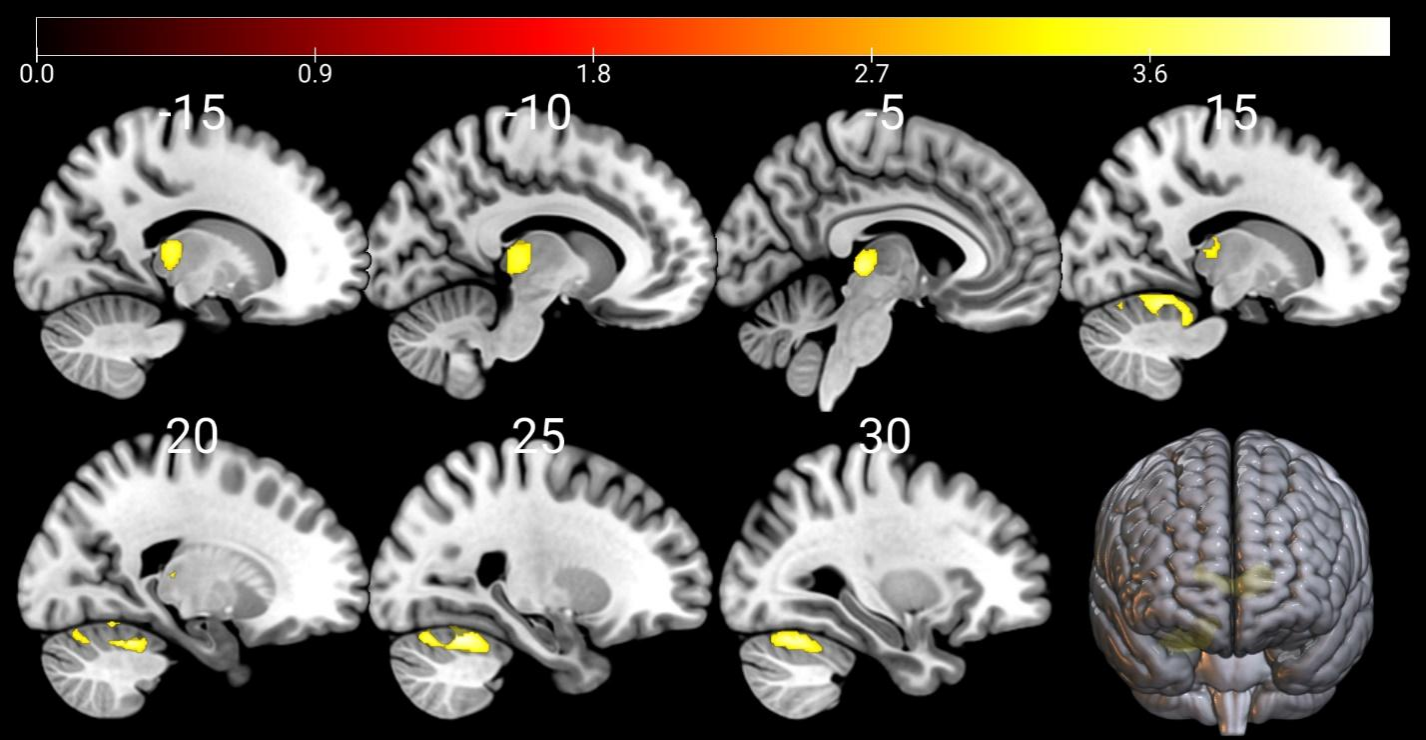
**Gray matter volume comparison by VBM among 52 PD-UNC patients compared to 192 PD-SNC**. PD-UNC patients exhibited a greater amount of GM in the cerebellar posterior and anterior lobes, as well as the declive, culmen regions of the vermis, sub-lobar, extra-nuclear, thalamus, and pulvinar, when compared to PD-SNC. Notable changes are shown within the figure as coloured regions. The map of *t*-values was generated via uncorrected contrast with a *p*<0.001 and an extent threshold of greater than 900 voxels. The scale of the *t*-statistic is given by the colour bar on the right-hand of the figure. Note that the vertical lines of the whole brain volume (bottom right) give the relative location of the visualized slices within the brain.

The ANOVA test comparing GM, CSF and TIV volumes among the HC, PD-UNC and PD-SNC groups were not significant (p > 0.09). The WM volumes were significantly different across groups (F (2, 346) = 3.67, p = 0.026), but subsequent post-hoc analyses did not uncover significant pair-wise differences (Fig. 1). The VBM analysis showed a significant increase in GM volume in the posterior and anterior lobes of the cerebellum, sub-lobar, extra-nuclear, thalamus and pulvinar regions among PD-UNC patients when compared to PD-SNC patients at baseline (Fig. 2 and Table 3). In contrast, no significant GM reduction was seen in PD-UNC patients when compared to PD-SNC. The VBM and DBM analyses revealed no significant differences between PD-UNC and PD-SNC in terms of WM or deep brain structures.

**Figure 3.**
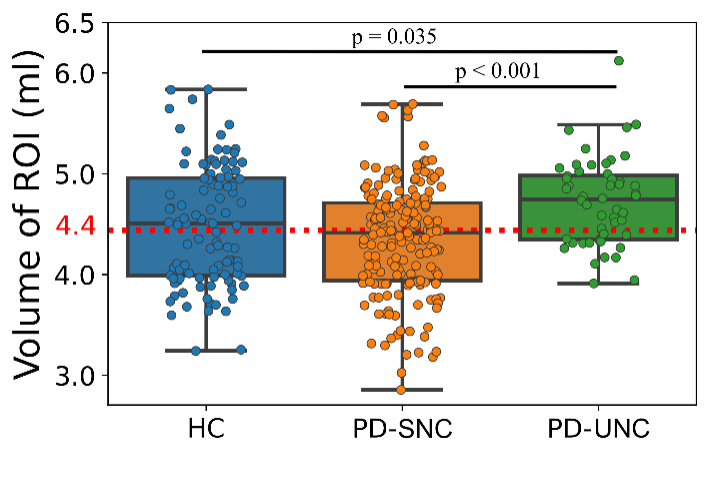
**Box plots displaying the adjusted ROI volumes in different groups**. Pairwise comparisons were conducted through an ANOVA test with *p*-value adjusted by Bonferroni correction. The HC group was used as a reference in volumetric analysis. The red dashed line represents the average GM volume among the three groups, which is 4.4 ml.

Mean GM volumes were extracted from identical clusters (Fig. 3) in all three groups to test the direction of GM volume differences compared to the HC group. To ensure a fair and unbiased comparison, we removed the effects of age, sex and differences in TIV through regression by using our independent group of healthy controls. ANOVA test revealed a statistically significant difference (F (2,346) = 9.76, p < 0.001; Fig. 3). The post-hoc analyses did not reveal significant differences between the HC and PD-SNC (mean difference = 0.13, p = 0.11, post-hoc Bonferroni). The PD-UNC group showed marginally significant higher GM volume than the HC group (mean difference = -0.23, p = 0.035, post-hoc Bonferroni). As hypothesized, there was a significant difference between PD-SNC and PD-UNC groups (mean difference = -0.37, p < 0.001, post-hoc Bonferroni), highlighting a notable distinction between these groups. These results were replicated when propensity score-matched PD-SNC* was used (mean ROI GM volume in PD-SNC* = 4.36 ± 0.61 ml; ANOVA: F (2,206) = 5.29, p = 0.006; post-hoc pairwise comparison between HC vs. PD-SNC*: mean difference = 0.11, p = 0.64, post-hoc Bonferroni; HC vs. PD-UNC: mean difference = -0.23, p = 0.045, post-hoc Bonferroni; PD-SNC* vs. PD-UNC: mean difference = -0.34, p = 0.005, post-hoc Bonferroni).

### Brain structural analysis: cortical thickness

No significant difference was observed between the two groups in terms of cortical thickness after controlling for age and sex (q > 0.20, FDR-corrected).

### Brain-PAD analysis

Our prediction model performed very well on the training (MAE = 4.72 years, RMSE = 6.07 years, R^2^ = 0.91, mean Brain-PAD = 0 ± 4.80 years) and hold-out HC sets (MAE = 4.63 years, RMSE = 5.88 years, R^2^ = 0.91, mean Brain-PAD = -0.08 ± 5.90 years) and in line with standards common within brain age prediction literature [30, 31]. Our prediction model did not show any sex bias in the training set (*t*(947) = 0.52, p = 0.60) or as the hold-out HC set (*t*(103) = 0.56, p = 0.57) with regard to brain-PAD. The mean Brain-PAD for all PD patients (*N_total_* = 244) used in this study was +3.11 ± 7.54 years, which was statistically higher than the hold-out HC group (t(347) = 3.86, p < 0.001). The mean Brain-PAD of +3.11 years for PD patients that we observed agrees with the previous studies [20, 21].

**Figure 4.**
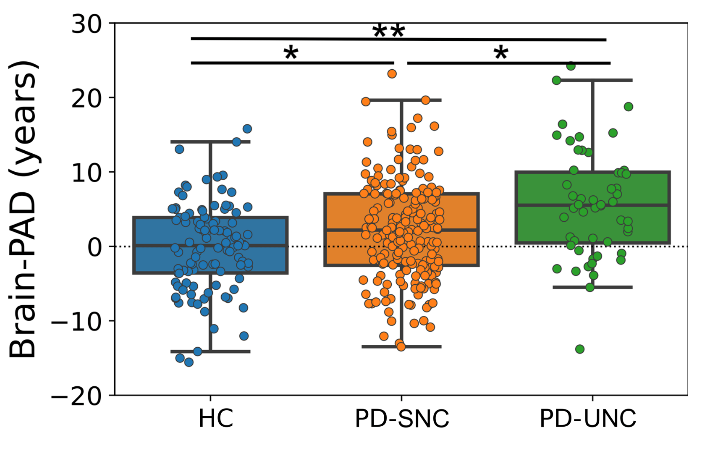
**Box plots displaying the Brain-PAD values in hold-out sets**. The mean Brain-PAD values of each group were depicted by a solid black line while the reference line (y = 0) was indicated by a dashed black line. Pairwise comparisons were conducted through an ANCOVA test adjusted for age and sex, with the *p*-value adjusted by Bonferroni correction. * indicates *p* < 0.05 and ** indicates *p* < 0.001.

Figure 4 shows the grouped data plots displacing the Brain-PAD in hold-out and PD sets. We performed ANCOVA testing to investigate any differences in mean Brain-PAD values among PD subjects with normal cognition at baseline and the HC group. The ANCOVA test revealed a significant difference in Brain-PAD among groups after accounting for sex and chronological age [F (2,344) = 9.33, *p* < 0.001). Real age did not show a significant effect on Brain-PAD (F (1,344) = 0.12, *p* = 0.72), indicating that the variation in Brain-PAD is not explained by differences in chronological age. The effect of sex on Brain-PAD was marginally significant (F (1,344) = 3.88, *p* = 0.049), suggesting that there may be some differences in Brain-PAD based on sex.

Both PD groups showed a significantly higher mean Brain-PAD (*p* < 0.02) compared to the HC group (Fig. 4). The PD-UNC group showed the highest Brain-PAD with a value of +5.66 ± 7.40 years. The mean Brain-PAD in PD-SNC was +2.42 ± 7.45 years. The Brain-PAD of the HC group was significantly different from both PD-SNC (mean difference = 2.38, *p* = 0.025, post-hoc Bonferroni) and PD-UNC group (mean difference = 5.49, *p* < 0.001, post-hoc Bonferroni). Brain-PAD was also significantly different between PD-SNC and PD-UNC (mean difference = 3.15, *p* = 0.014, post-hoc Bonferroni). This finding was replicated when PD-SNC* was used and ANCOVA testing revealed a significant difference after adjusting for sex and chronological age (F (2,204) = 8.77, p < 0.001). Real age (F (1,204) = 0.14, p = 0.70) and sex (F (1,204) = 3.34, p = 0.07) did not exhibit a significant effect on Brain-PAD. Mean Brain-PAD in PD-SNC* was +2.37 ± 8.35 years. Brain-PAD of the HC group significantly differed from both PD-SNC* (mean difference = 2.18, p = 0.31, post-hoc Bonferroni) and PD-UNC group (mean difference = 5.75, p < 0.001, post-hoc Bonferroni). Finally, Brain-PAD showed a significant difference between PD-SNC* and PD-UNC (mean difference = 3.30, p = 0.047, post-hoc Bonferroni).

### The relationship between GM of ROI and Brain-PAD

**Figure 5.**
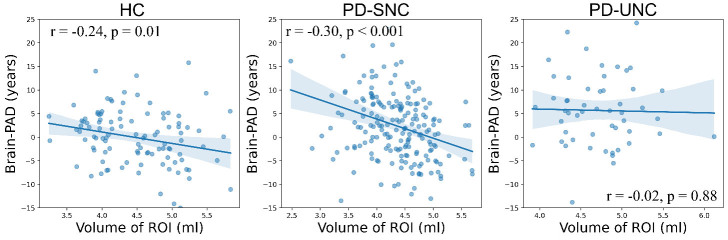
**The correlation between adjusted GM volumes in the region that was identified by VBM between PD-SNC and PD-UNC groups, and Brain-PAD within each group, adjusting for sex, real age and TIV**. The HC group was used as a reference in this analysis.

Figure 5 illustrates the relationship between adjusted GM volumes in the cluster identified from contrasting PD-SNC vs. PD-UNC (Fig. 2) and Brain-PAD within each group. The HC group showed a moderate negative correlation between this regional GM volume and Brain-PAD (*r* = -0.241, *p* = 0.01), demonstrating that a decreased GM volume in this region is associated with accelerated brain aging among HC subjects. A similar pattern was observed in the PD-SNC group (*r* = -0.302, *p* <0.001) and in PD-SNC* (r = -0.50 and p< 0.001), which further supports the relationship between regional GM volume and Brain-PAD. Patients within PD-UNC did not demonstrate this pattern (*r* = -0.02, *p* = 0.88), suggesting a potential disturbance on the role of cerebellum in brain aging in PD patients who later experience cognitive decline.

**Table 3:** The brain regions with increased GM volume in 52 PD patients who transitioned from normal cognition to mild cognitive impairment compared to 192 PD patients who maintained normal cognitive status.

Cluster	Region	Cluster Size (No. of Voxels)	q (FDR)	Hemisphere	MNI Coordinates (x, y, z)	*t*-Value (Peak Voxel)
1	Right Cerebellum/ Cerebellum Anterior Lobe / Culmen /Cerebellum Posterior Lobe/ Declive	1271	0.016	R	25, -57, -21	4.43
2	Left Cerebrum/Sub-lobar/ Extra-Nuclear/ Thalamus/ Pulvinar	931	0.046	L	-3, -27, 2	4.03

R = right hemisphere = Montreal Neurological Institute; FDR = false discovery rate.

### Clinical correlates of imaging-based findings

**Figure 6.**
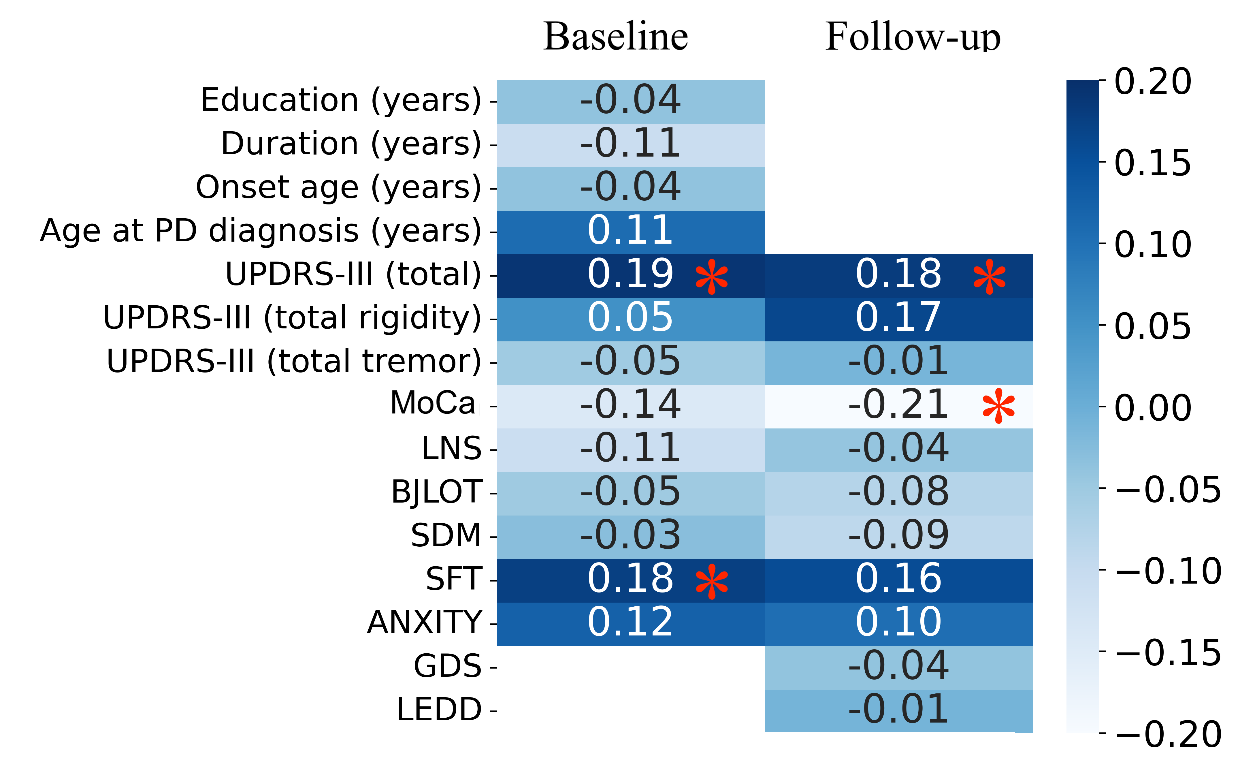
**The relationship between Brain-PAD measured at baseline and patient features in both groups was assessed using Pearson correlation test for continuous variables and Spearman rank correlation test for discrete variables**. The p-values were corrected using FDR correction for multiple comparisons. The red star displayed in the boxed area indicates a significant correlation with Brain-PAD (*P < 0.05). The color bar and numbers in the boxes represent correlation values.

A significant association was found between Brain-PAD and UPDRS III (total) at baseline and follow-up, MoCA scores at follow-up, as well as SFT at baseline (Fig. 6) in both groups (N=244). There was, however, no association between ROI GM volume and clinical variables in either group. Further analysis within each group revealed a significant association between Brain-PAD and LNS at follow-up in PD-SNC (r= -0.23, p= 0.02, after FDR correction). Time to conversion from HC to MCI for PD-UNC subjects was not significantly associated with GM ROI volume (r = -0.09, p = -.49) nor Brain-PAD (r= -0.21, p = 0.059) at baseline.

## DISCUSSION

Our goal for this study was to investigate the existence of any early neuroanatomical signature for the progression from normal cognition to MCI in patients with PD. To this end we evaluated neuroimaging data for patient groups using VBM and brain age estimation techniques. Our VBM analysis revealed for the first time that PD-UNC patients had greater GM volumes in the cerebellar posterior and anterior lobes, the vermis (decline and culmen regions), and also the sub-lobar, extra-nuclear, thalamus and pulvinar regions compared to PD-SNC patients at baseline (Fig. 2 and Table 3). Interestingly, GM volumes extracted from identical clusters of HC group were greater than in PD-SNC but lower for PD-UHC. This result potentially suggests regional ROI GM volume differences found in PD patients. The GM volumes of PD-SNC did not show significant deviation from normal ranges, unlike PD-UNC (Fig 3). Decreased GM volumes are typically associated with atrophy, however we stress that the etiology of increased GM volume in neurodegenerative disorders are not well understood [32]. Nevertheless, increased cerebellar glucose metabolism has consistently been observed in the literature in cognitively impaired PD patients [33, 34], which is frequently hypothesized to be related with compensatory mechanisms. Structural biomarkers associated with cognitive decline in PD (i.e., in PD-MCI group) have been reported in temporal-parietal and basal forebrain [35-37]. VBM studies have also documented significant GM decreases in the right insula, right inferior frontal gyrus, middle frontal gyrus and right cerebellum in PD-MCI patients compared to cognitively healthy PD patients [38].

It is noteworthy that we observed an increase in GM within the cerebellum, sub-lobar, extra-nuclear, thalamus and pulvinar regions of PD-UNC patients compared to PD-SNC before they exhibited noticeable cognitive symptoms. This finding suggests that GM abnormalities in these regions (particularly the cerebellum) may be a distinctive feature in the very early stages of cognitive decline in PD. This finding remained stable even after incorporating motor (UPDRS-III) and non-motor (MoCA) variables as covariates, along with age, sex and TIV into the VBM analysis. This suggests that the gray matter abnormalities in these regions are potentially independent of subtle clinical differences between the two groups. The role of cerebellum has traditionally been viewed as controlling and harmonizing voluntary movement, balance, posture and motor learning. Cerebellar abnormalities have traditionally been implicated in the tremor [39] and gait disturbances [40]. More recently, it has been hypothesized that cerebellum is also involved in cognitive decline shown in PD [40, 41]. Previously, increased cerebellar glucose metabolism has reported to be related with cognitive decline in PD [34]. On the contrary, cerebellar atrophy has been associated with patients’ impaired performance on semantic fluency tests [42] and executive function [43], potentially suggesting the increased cerebellar metabolism may reflect compensatory mechanisms, although our previous graph theory analysis suggested denser connections within the hyper-metabolic regions including limbic-pontine-cerebellar network in PD is the key contributor for the formation of the overall PD-related metabolic pattern that characterizes PD brain’s hyper-smallworldness [44].

Morphological changes in brain WM have been linked to MCI in PD, such as reduced WM density in the midbrain, occipital lobe, inferior frontal gyrus and lingual gyrus in PD-MCI patients compared to PD patients with normal cognition [45]. Our VBM analysis on WM did not identify any significant differences between the PD-SNC and PD-UNC groups, suggesting that both groups had no change in WM or comparable changes in WM at baseline. The results of the DBM analysis also did not indicate any significant variation between the PD-SNC and PD-UNC groups. It has been suggested that DBM is more sensitive in detecting brain atrophy in subcortical regions than VBM, and it has been recently used to detect the brain abnormalities in PD [46, 47], however, it is yet unknown if DBM is sensitive to GM volume increase as identified in our study.

We also performed a cortical thickness analysis, which has been suggested to be more sensitive than VBM in detecting regional gray matter changes associated with the disease [48, 49]. Compared to PD patients without MCI, PD-MCI patients showed reduced cortical thickness in the medial temporal, superior frontal, inferior parietal and supramarginal gyri, the precentral gyrus, the precuneus, the insula and the occipital cortex regions [38, 50, 51]. In our analysis, we did not observe any distinctions between the PD-SNC and PD-UNC groups in cortical thickness, implying that both groups experience a similar rate of cortical thinning at baseline. It should also be noted the cortical thickness analysis result can vary depending on the atlases that were used. The DKT atlas that we used is optimized for cerebral cortex and its performance in thalamic and cerebellar has not been validated rigorously.

It should be noted that our negative findings do not contradict the previous positive neuroimaging studies [38] who cross-sectionally contrasted PD-MCI with PD patients with normal cognition. This is so because both our PD-UNC and PD-SNC were cognitively normal at the time of MRI imaging. Rather, our study results imply that previously reported atrophy at the MCI stage simply may not precede cognitive decline.

Using our brain age estimation model, we replicated the overall increase of Brain-PAD in our PD patients compared to HC (Fig. 4) [20]. In the PD group, increased Brain-PAD has been associated with a decrease in GM volume in the limbic, occipital, temporal, parietal and primary frontal lobes, as well as a decrease in WM volume in the frontal lobe, cerebellum, midbrain, lentiform nucleus and medulla. We found that the Brain-PAD was significantly higher in PD-UNC patients than PD-SNC patients (Fig. 4). This result support our hypothesis that PD-UNC patients experience an accelerated brain atrophy linked to aging at baseline compared to PD-SNC. This result is in line with previous VBM studies contrasting PD patients with and without MCI [52, 53], while highlighting that overall brain atrophy associated with normal aging process was more accelerated in PD-UNC patients even at baseline when they still showed normal cognition.

Significant inverse correlations were observed between Brain-PAD and the ROI GM volumes in both the HC and PD-SNC groups, suggesting that the observed atrophy in these regions (Table 3) is associated with accelerated brain aging processes. Interestingly, the PD-UNC patients did not exhibit the same pattern (Fig 5). One potential explanation for this discrepancy is that the enlarged GM volumes (and/or hypermetabolism as previously reported [40]) in the identified regions (particularly in the cerebellum) may signify a reaction to disease pathology, compensatory neural hypertrophy, neuroinflammation, or a blend of these factors and other processes that are presently not well comprehended. Our interpretation may be consistent with previous research indicating that increased cortical thickness/volume is linked to initial cognitive decline in cognitively normal adults and the initial phases of Alzheimer's disease pathology [54].

An important limitation to the presented study is the limited sample size in the PD-UNC group. A similar limitation is using only baseline MRI studies without incorporating longitudinally acquired MRI studies. PPMI data collection is not yet complete but is expected to be so in the near future. The inclusion of additional data, especially an expected sizable increase in the number of PD patients who progress to dementia, will be of significant interest for further work on this topic. This prospective increase in data availability will allow the development of a more comprehensive model to predict cognitive decline in PD. We also note that the interpretation of our results and comparison with the existing literature should be carefully considered in view of the substantial variability in data selection and sources, pre-processing procedures, choice of statistical model and validation procedures employed across different studies.

### Conclusion

This study provides the first empirical evidence that there are distinct structural brain differences between PD-SNC and PD-UNC at baseline. Using VBM, we identified an increased GM volume in the cerebellar regions of PD-UNC patients compared to PD-SNC patients. This intriguing finding suggests that the cerebellum may play a compensatory role in the very early stage of cognitive decline in PD patients’ years before symptoms become clinically evaluable. We also provided evidence that patients with PD will transition from normal cognitive function to MCI have a greater Brain-PAD than those with stable cognition. These findings provide valuable insights into the origins of cognitive impairment in the earliest stages of PD and also offer prospects for future interventions targeting PD patients who are at risk of cognitive decline by introducing a data-driven biomarker that may enrich future trials targeting preventative therapies.

## Data Availability

The raw MRI scans used in this study are publicly available at Open Access Series of Imaging Studies (OASIS; https://www.oasis-brains.org/), the IXI (https://brain-development.org/ixi-dataset/), and Parkinson’s Progression Markers Initiative (PPMI) databases. Approval from an ethics committee for human experimentation was obtained for each database prior to the initiation of the study, and written informed consent was provided by the participants.

## Code Availability

We performed brain age estimation and bias adjustment on the basis of our previously validated framework, which can be found on https://github.com/medicslab/Bias_Correction. The code utilized for brain age estimation in this study is available upon request.
